# HybridSense-LLM: A Structured Multimodal Framework for Large-Language-Model–Based Wellness Prediction from Wearable Sensors with Contextual Self-Reports

**DOI:** 10.3390/bioengineering13010120

**Published:** 2026-01-20

**Authors:** Cheng-Huan Yu, Mohammad Masum

**Affiliations:** Department of Applied Data Science, San Jose State University, San Jose, CA 95192, USA; cheng-huan.yu@sjsu.edu

**Keywords:** large language models (LLMs), wearable sensor analytics, wellness prediction, hybrid multimodal representations, prompt engineering, physiological signal modeling, digital health monitoring

## Abstract

Wearable sensors generate continuous physiological and behavioral data at a population scale, yet wellness prediction remains limited by noisy measurements, irregular sampling, and subjective outcomes. We introduce HybridSense, a unified framework that integrates raw wearable signals and their statistical descriptors with large language model–based reasoning to produce accurate and interpretable estimates of stress, fatigue, readiness, and sleep quality. Using the PMData dataset, minute-level heart rate and activity logs are transformed into daily statistical features, whose relevance is ranked using a Random Forest model. These features, together with short waveform segments, are embedded into structured prompts and evaluated across seven prompting strategies using three large language model families: OpenAI 4o-mini, Gemini 2.0 Flash, and DeepSeek Chat. Bootstrap analyses demonstrate robust, task-dependent performance. Zero-shot prompting performs best for fatigue and stress, while few-shot prompting improves sleep-quality estimation. HybridSense further enhances readiness prediction by combining high-level descriptors with waveform context, and self-consistency and tree-of-thought prompting stabilize predictions for highly variable targets. All evaluated models exhibit low inference cost and practical latency. These results suggest that prompt-driven large language model reasoning, when paired with interpretable signal features, offers a scalable and transparent approach to wellness prediction from consumer wearable data.

## 1. Introduction

Wearable devices such as Fitbit, Apple Watch, Samsung Galaxy Watch, Garmin, WHOOP, and Oura Ring now collect continuous physiological and behavioral data at a population scale. Approximately 45% of American adults use these devices to monitor heart rate, physical activity, sleep duration, and related indicators, creating a substantial foundation for personalized wellness assessment [1]. Prior work demonstrates that wearable-derived signals can support prediction of stress, fatigue, readiness, and sleep quality [2,3,4,5]. However, free-living wearable data are inherently noisy, irregularly sampled, and strongly shaped by individual behavior and context, which complicates robust modeling [6,7,8]. In addition, wellness outcomes are typically obtained through subjective self-reports that lack standardized reference scales, limiting the reliability and generalizability of traditional machine learning approaches [9,10].

Recent advances in generative artificial intelligence suggest that large language models offer a complementary paradigm for wellness analytics by reasoning over heterogeneous numerical and contextual inputs. LLMs have achieved strong performance in large-scale clinical prediction from electronic health records [11] and have shown the ability to interpret engineered wearable descriptors when guided by structured prompts [12]. Progress in time-series LLMs further demonstrates that numerical sequences can be reformatted into language-aligned representations, enabling models to reason over temporal structure without retraining [13,14,15,16,17]. At the same time, prompt-engineering studies indicate that reasoning style and task framing substantially influence predictive accuracy in health applications [12]. Despite this progress, existing work does not examine how statistical features and raw signal segments should be jointly structured for LLM reasoning, nor does it systematically compare prompting strategies across multiple wellness targets.

To address these gaps, we introduce HybridSense, a framework that integrates raw wearable signal segments and their associated statistical descriptors with LLM-based reasoning for daily wellness prediction. Wearable time series are transformed into descriptive statistical features, key predictors are identified using a Random Forest model, and both feature-level and waveform-level information are embedded into standardized prompts. We evaluate seven prompting strategies across three LLM families and four wellness targets, supported by bootstrap analyses to assess accuracy, stability, and computational efficiency. Accurate estimation of stress, fatigue, readiness, and sleep quality has direct implications for preventive health, athletic training, occupational safety, and personalized behavioral support [18,19,20,21].

This work builds on advances in wearable time-series representation learning and multimodal digital health. Deep learning models increasingly fuse accelerometer, photoplethysmography, and temperature signals to infer fatigue, stress, and circadian disruption in free-living settings [22,23,24]. However, wearable data are often variable in length, incomplete, and highly individualized, motivating embedding-based approaches such as HeartSpace, which learns robust representations from variable-length and missing signals via temporal encoding and pattern aggregation [25]. Complementary work shows that clustering contextual subsequence embeddings reduces dispersion and improves model-independent time-series prediction accuracy [26].

In parallel, large language models are emerging as flexible interfaces for interpreting complex wearable sensor streams, despite challenges in scale, heterogeneity, and temporal structure [27]. Clinical studies further highlight the need for multimodal integration, explainable AI, and deployment-ready frameworks to translate wearable analytics into practice [28]. Recent advances in edge intelligence and deep-learning–enabled edge computing further emphasize the importance of deployment-ready, low-latency, and resource-efficient architectures for translating multimodal wearable analytics into real-world clinical and consumer applications [29,30]. Meanwhile, progress in biomedical generative models, including BioGPT [31], MedAlpaca [32], Meditron [33], ClinicalCamel [34], and hybrid architectures such as BioMedGPT [35] and GatorTronGPT [36], underscores the potential of language-based reasoning to augment numerical sensing. Recent studies further show that prompt-tuned and retrieval-augmented LLMs can support time-series interpretation, explanation, and personalized coaching in wearable contexts [37,38,39,40,41]. Together, these developments motivate HybridSense as a principled framework for language-driven reasoning over continuous wearable data using structured statistical and signal-level representations.

The main contributions of this work are as follows:

- We introduce HybridSense, a framework that integrates raw wearable signal segments and their statistical descriptors with LLM-based reasoning for daily wellness prediction.
- We systematically compare seven prompting strategies across four wellness targets and identify clear task-dependent performance patterns using 30-round bootstrap analysis.
- We develop two complementary input representations, including a statistical descriptor model and a hybrid model that improves readiness estimation by combining features with waveform context.
- We evaluate three major LLM families and quantify inference cost and latency, demonstrating the practical feasibility of LLM-driven wellness prediction.

## 2. Methods

### 2.1. Framework Overview

**Figure 1:**
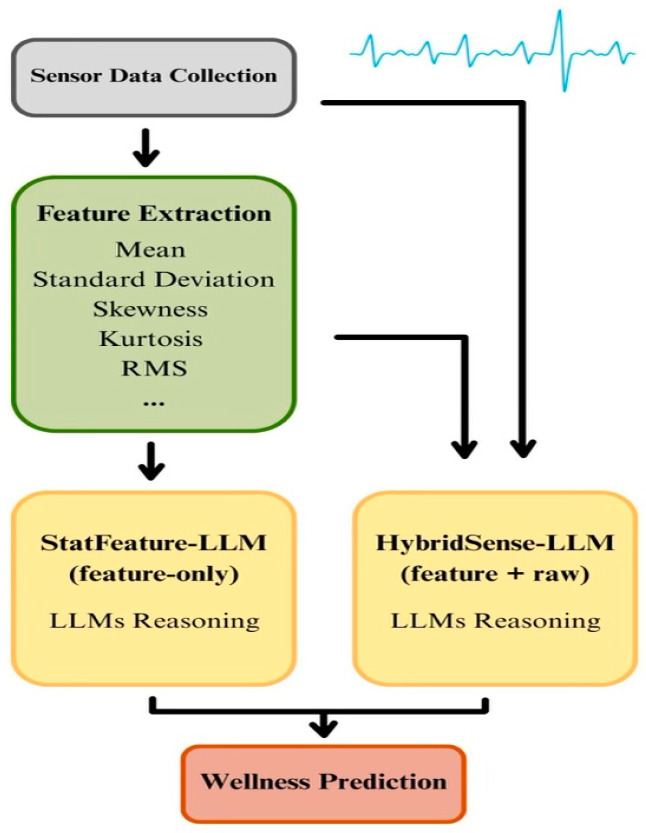
The diagram illustrates how daily wearable sensor data are transformed into statistical features and optionally combined with raw signal segments before being processed by StatFeature-LLM or HybridSense-LLM to generate predictions for daily wellness states.

HybridSense is a multimodal framework that integrates wearable-derived statistical descriptors, optional raw signal segments, and structured large language model reasoning to estimate daily wellness states. The pipeline transforms heterogeneous physiological signals into interpretable prompt-based representations and evaluates how different reasoning strategies influence prediction accuracy. The framework consists of four stages: feature extraction, feature selection, prompt construction, and model evaluation, as illustrated in Figure 1.

### 2.2. Feature Extraction and Selection

Raw wearable streams, including heart rate, step count, and activity intensity, are preprocessed and aligned to a daily resolution. From each daily segment, statistical descriptors are computed to capture central tendency, variability, and distributional characteristics, including mean, standard deviation, skewness, kurtosis, and related metrics. These descriptors provide a compact numerical summary of short-term physiological patterns. Feature relevance is assessed using Random Forest regression models trained separately for each wellness target. Feature importance is computed using impurity-based importance, defined as the mean decrease in variance across tree splits, as implemented in the scikit-learn Random Forest Regressor library. Importance scores are averaged across trees and normalized within each target. Features are ranked accordingly, and the smallest subset whose cumulative importance exceeds 80 percent is retained for downstream analysis. No permutation-based or coverage-based importance methods are used.

### 2.3. Prompt Construction and LLM Configurations

Two large language model configurations are evaluated. StatFeature-LLM receives only the selected statistical descriptors as input. HybridSense-LLM extends this representation by additionally including short raw waveform segments within the same structured prompt. The two configurations are evaluated independently and are not ensembled or numerically combined, ensuring that observed performance differences reflect input representation rather than post hoc fusion.

Each prompt specifies the prediction target, presents inputs in a standardized tabular format, and constrains the model output to a single numeric value. Seven prompting strategies are evaluated: zero-shot, chain of thought, tree of thought, least-to-most, self-consistency, few-shot, and few-shot with reasoning. Table 1 summarizes the reasoning mechanisms underlying these strategies, and Table 2 details the prompt template components used to ensure consistent formatting and output constraints.

### 2.4. Output Validation, Parsing, and Evaluation

Large language model outputs are required to return a single scalar value per target per day. Responses are validated by enforcing numeric format compliance and valid value ranges. Valid outputs are deterministically parsed to extract the predicted scalar score. Predictions are temporally aligned with self-reported wellness labels using day-level matching for each participant.

Model performance is evaluated using mean absolute error, computed between the predicted scalar score and the corresponding self-reported label for the same participant and day. In addition to aggregate error metrics, temporal alignment between predicted and reported wellness trajectories is examined by comparing day-to-day trends within participants. Robustness is assessed using bootstrap resampling, in which 50 percent of daily records are sampled with replacement over 30 iterations to estimate stability.

## 3. Experimental Design

### 3.1. Dataset Description

This study uses the public PMData dataset, which contains multi-week recordings from 16 adult participants wearing Fitbit Versa 2 smartwatches in naturalistic settings. The dataset integrates three complementary information streams: minute-level logs of heart rate and step count, daily summaries such as total steps, calories burned, active minutes, and resting heart rate, and daily self-reported wellness scores describing mood, stress, fatigue, readiness, and sleep quality. All self-reports use five-point Likert scales except readiness, which ranges from 0 to 10.

Although mood is collected daily alongside other wellness measures, it is treated in this study as a contextual behavioral input rather than a prediction target. This follows prior LLM-based wellness frameworks, such as Health-LLM, where self-reported affective states are used as auxiliary context to support reasoning over downstream wellness outcomes instead of being optimized as primary targets [12].

To construct a consistent analytical panel, all CSV and JSON files are merged by participant ID and timestamp, and each calendar day is treated as a single observation. From the raw physiological and activity signals, we compute descriptive statistics including mean, minimum, maximum, standard deviation, skewness, kurtosis, root mean square, and interquartile range. These descriptors summarize both the magnitude and variability of daily physiological behavior. After preprocessing, the dataset contains 1836 daily records with approximately 25 to 30 numerical features and four target variables.

Missing data arising from device non-wear or incomplete logging are handled through strict data-quality filtering rather than statistical imputation. Hourly wearable measurements are first aggregated to daily summaries using standard statistical operators. For downstream analysis, only consecutive 7-day windows with complete daily records are retained; if any single day within a 7-day window is missing, the entire window is excluded from modeling. As a result, no mean, median, or mode imputation is applied at either the hourly or daily level.

The dataset includes five key behavioral and physiological indicators recorded at different temporal resolutions. Step count and calorie expenditure are collected at a five-minute cadence and aggregated to daily summaries, while resting heart rate and sleep duration are provided as daily measures. Mood is recorded once per day through self-report. All variables are aligned by calendar date and represented at a daily resolution to ensure consistency with the wellness prediction targets.

The raw wearable signals and their transformation into daily predictors, along with participant characteristics, data volume, feature composition, and preprocessing steps, are summarized in the Supplementary Materials (Tables S1–S4).

### 3.2. Data Processing and Feature Engineering

The PMData files contain heterogeneous sensor streams collected at different temporal resolutions, including minute-level heart rate and activity logs, daily behavioral summaries, and self-reported wellness scores. To create a unified analytical structure, all signals are aligned to a daily cadence. High-frequency streams are aggregated using descriptive statistics such as mean, standard deviation, and range, producing one complete daily observation per participant (Tables S1–S3).

To capture short-term temporal structure while maintaining consistency with daily wellness annotations, we apply a sliding-window approach that groups consecutive days into fixed segments. Only windows containing complete daily records are retained. Target scores for readiness, stress, fatigue, and sleep quality are averaged within each window, reducing subjective noise and emphasizing stable behavioral trends. From each windowed segment, we compute a comprehensive set of statistical descriptors, including mean, minimum, maximum, skewness, kurtosis, root mean square, interquartile range, coefficient of variation, and peak-to-peak range (Table S4). These descriptors provide an interpretable representation of day-level physiological dispersion and behavioral patterns.

The resulting n-by-k feature matrix captures both inter-individual differences and intra-individual temporal fluctuations. To identify the most informative predictors, we train a Random Forest regressor for each wellness target and retain the smallest subset of features whose cumulative importance reaches 80 percent. This selection step reduces redundancy, improves computational efficiency, and highlights the physiological and behavioral indicators most strongly associated with day-level wellness states. Table 3 presents the top predictive features for fatigue, readiness, sleep quality, and stress. Mood-derived descriptors are included as contextual self-reported features to capture short-term affective dynamics and are not treated as outcome variables in any prediction task.

Across all four wellness targets, mood-related descriptors consistently rank among the strongest predictors, highlighting the close association between emotional dynamics and daily physiological state. Readiness and stress are particularly influenced by mood-dispersion metrics, while sleep quality and fatigue additionally depend on sleep-duration statistics and day-level heart-rate summary descriptors. Activity-based features, such as step-count and calorie-burn variability, provide complementary behavioral context but play a secondary role. Together, these patterns indicate that short-term emotional dynamics, combined with cardiovascular and sleep stability at the day level, provide reliable signals for estimating wellness states from consumer wearable data.

Overall, these processing steps transform noisy, heterogeneous wearable logs into a structured and information-dense feature space that supports rigorous and interpretable evaluation of the HybridSense framework.

### 3.3. Input Representations, Prompt Design, and Evaluation Protocol

To evaluate how input representation influences prediction performance, we consider two complementary LLM configurations that differ only in the information provided to the model. StatFeature-LLM receives a compact set of engineered statistical descriptors derived from wearable signals, including measures of central tendency, dispersion, and distributional shape. These descriptors provide an interpretable, low-noise summary of daily physiological activity and follow established feature-engineering practice in wearable and physiological signal modeling.

HybridSense-LLM extends this representation by augmenting the same statistical descriptors with short raw sensor segments drawn from heart rate, activity intensity, and motion signals. This hybrid input preserves localized temporal fluctuations and micro-variations that are inherently lost during full aggregation. Intuitively, StatFeature-LLM characterizes a day using summary numbers such as average heart rate or overall variability, whereas HybridSense-LLM additionally presents a brief snapshot of how those signals evolve within the day. This allows the model to reason jointly over global trends and fine-grained temporal structure without retraining or modifying the underlying language model.

Both configurations use identical prompt templates and evaluation procedures, ensuring that observed performance differences reflect input representation rather than prompting or model effects. This controlled comparison isolates the contribution of hybrid statistical and signal-level inputs to LLM-based wellness prediction.

All prompts follow a common structure:

- Information/context: A concise description of available inputs and their sources (for example, Fitbit-derived features and self-reports), including variable names and units.
- Health context: A brief rationale linking the target variable (readiness, stress, fatigue, or sleep quality) to physiological or behavioral markers and its relevance for daily wellness monitoring.
- Instruction: Clear, actionable steps that specify how the model should analyze the features, reason internally, and produce a single score within a specified range.
- Output format: An exact specification of the required output, such as “Return one real number in [0,5] with one decimal; no explanations.”
- Example: A small input–output illustration showing feature values and the correctly formatted response.

This template is flexible across wellness targets while maintaining a consistent structure to ensure fair comparison across prompting strategies. Readiness predictions are reported on a 0–10 scale, whereas stress, fatigue, and sleep quality are reported on a 0–5 scale. The template is instantiated for seven distinct prompting strategies. Concrete prompt examples for daily stress prediction, including task instructions and mood-related statistical inputs such as Mood_skewness, Mood_std, and Mood_cv, are provided in the Supplementary Materials (Tables S5–S11), where the model is framed as a health-focused AI estimating stress from physiological and behavioral indicators.

To support large-scale experimentation, we implement an automated batch system that handles prompt generation, LLM calls, timeout detection, retry logic, and result storage. All outputs are parsed and validated to ensure that predicted scores are numeric and within the correct range for the corresponding target. The experiments use OpenAI GPT-4o-mini, Gemini, and DeepSeek models through their APIs, with settings chosen to minimize randomness and improve consistency. This framework yields a reproducible and scalable evaluation protocol for comparing input configurations and prompting strategies in LLM-based wellness prediction.

### 3.4. Large Language Models and Inference Configuration

All experiments were conducted under fully specified and controlled inference settings to ensure reproducibility. Three commercial LLM families were evaluated: OpenAI GPT-4o-mini, Google Gemini 2.0 Flash, and DeepSeek Chat. All models were accessed exclusively through their official public APIs and used strictly in inference mode, with no fine-tuning, parameter updates, or task-specific training on the PMData dataset or related wearable data.

OpenAI-based inference used the GPT-4o-mini model via the Chat Completions API. Each request consisted of a single user-role message containing the constructed prompt, without system instructions, tool calls, or conversational memory. The decoding temperature was fixed to 0.0 to enforce deterministic generation, while all other decoding parameters, including nucleus sampling, frequency penalty, and presence penalty, were left at default values. Each request returned a single completion, from which the first valid floating-point number was extracted using a regular-expression parser and used as the prediction.

DeepSeek inference used the deepseek-chat model through a DeepSeek-compatible OpenAI client with a custom API base URL. The configuration matched the OpenAI setup, using a single user message per request, temperature set to 0.0, deterministic decoding, and one completion per prompt. Outputs were processed using the same numerical extraction procedure and clipped to the valid range of each wellness target. No additional reasoning flags, sampling heuristics, or adaptive decoding strategies were enabled.

Google Gemini inference was performed using the gemini-2.0-flash model via the google-generativeai SDK. The model instance was initialized once and reused across inference calls. Each request consisted of a single prompt string with temperature fixed to 0.0 and all other decoding parameters at default values. To limit nondeterminism from concurrent execution, parallel inference was restricted to two worker threads, and a fixed random seed was used for bootstrap sampling.

Across all models, prompts were generated deterministically from identical feature representations, task definitions, and reasoning templates, with no manual intervention or prompt modification between runs. Prompts explicitly constrained outputs to a single numeric value within the valid range of the target wellness score, and invalid outputs were discarded and regenerated using the same prompt.

## 4. Experimental Results

### 4.1. Performance Comparison Across Prompting Strategies and LLM Configurations

We evaluated all prompting strategies using a standardized bootstrap protocol (30 replications, 50 percent resampling with replacement). Table 4, Table 5 and Table 6 report mean absolute error (MAE) with bootstrap standard error (SE) for OpenAI 4o mini, Gemini 2.0 Flash, and DeepSeek-Chat. Across all models and targets, SE values remain small, confirming that performance estimates are stable and not driven by sampling noise.

Clear and consistent performance patterns emerge across prompting strategies and model families. OpenAI 4o mini exhibits the most stable behavior, showing clear separation between effective and ineffective prompting strategies. Gemini 2.0 Flash demonstrates greater variability and occasionally reverses trends observed elsewhere. DeepSeek-Chat displays strong accuracy overall, particularly under the HybridSense configuration, which incorporates both engineered features and raw waveform segments. Across all models, L2M remains the least effective strategy, while Zero-shot, Self-Consistency, and Few-shot+CoT emerge as the strongest baselines depending on the prediction target.

Fatigue Prediction: Across all three models, Zero-shot prompting provides the most reliable baseline for fatigue prediction. DeepSeek-Chat benefits noticeably from the HybridSense input design, achieving MAE = 0.34, which narrows the gap with Gemini and OpenAI. Complex reasoning strategies such as CoT and ToT increase error, suggesting that fatigue is best modeled through direct feature–response relationships rather than multi-stage reasoning. L2M consistently performs worst, indicating that decomposing the task into smaller subtasks does not align with the underlying physiological signal dynamics.

Readiness Prediction: Readiness demonstrates greater sensitivity to model choice and input representation. OpenAI 4o mini achieves the lowest MAE overall (0.535 under HybridSense), while DeepSeek-Chat attains competitive performance when waveform information is included (MAE = 0.73). Gemini 2.0 Flash shows notable instability for CoT and L2M, reflecting its sensitivity to deeper reasoning instructions. Overall, minimal-context strategies such as Zero-shot and ToT outperform multi-stage reasoning, indicating that readiness is better captured through direct interpretation of the combined statistical and waveform signals.

Sleep-Quality Prediction: Sleep quality benefits consistently from Few-shot and Few-shot+CoT prompting, which outperform all other strategies across model families. OpenAI 4o mini provides the most stable advantage (MAE ≈ 0.39), while Gemini exhibits wider dispersion, particularly under SC and ToT. DeepSeek-Chat produces mid-range and reliable estimates (0.39–0.48), suggesting that modest exemplar-based prompting helps the model navigate variability in subjective wellness scoring. These trends indicate that sleep quality, a more subjective and noisier target, maps better to exemplar-driven reasoning rather than rule-based instructions.

Stress Prediction: Stress prediction displays the strongest benefit from Self-Consistency, which delivers the lowest and most stable errors across models. OpenAI’s SC baseline achieves MAE = 0.32, and DeepSeek-Chat closely replicates this trend (≈0.32). Reasoning-oriented methods such as CoT and ToT do not produce meaningful improvements. L2M again performs worst, highlighting its sensitivity to label variance and the difficulty of decomposing stress into small independent subtasks. These findings imply that stress estimation benefits from ensembling multiple internal reasoning paths rather than following sequential reasoning workflows.

A cross-model comparison reveals three key observations:

- OpenAI 4o mini is the most stable and data-efficient, consistently outperforming or matching other systems across all targets and strategies.
- Gemini 2.0 Flash shows higher variance, benefiting occasionally from deeper reasoning (for example, CoT in sleep quality) but often producing inconsistent patterns, especially under L2M and multi-step prompts.
- DeepSeek-Chat excels under HybridSense, demonstrating strong ability to integrate raw waveform segments and engineered features. Its competitive performance suggests that multimodal sensitivity is a defining strength of the model.

Taken together, these results indicate that prompt design matters more than the choice of model family. Tasks with stable physiological signals (fatigue, readiness) favor simpler prompting, while more subjective targets (sleep quality, stress) benefit from exemplar-based or internally averaged reasoning (Few-shot, SC). HybridSense consistently improves performance for models capable of integrating waveform cues, such as DeepSeek-Chat.

### 4.2. Computational Cost and Inference Latency Evaluation

To assess the practical feasibility of deploying LLM-based wellness prediction in real-world monitoring systems, we evaluated both computational cost and inference latency using the same 30-bootstrap-per-target protocol applied in the performance experiments. Each bootstrap iteration represents a full prompt–response cycle for a single wellness target and mirrors the repeated daily evaluations expected in longitudinal health-monitoring settings.

All models were accessed through their API interfaces, and each bootstrap included prompt construction, model inference, postprocessing, and numerical validation. Latency was defined as the total elapsed time required to complete 30 replications for one target variable. Costs were computed using published token pricing, normalized to the average prompt and response length. Table 7 reports the resulting per-target cost and runtime. For Gemini 2.0 Flash, latency estimates were interpolated using observed inference throughput and pricing tiers comparable to OpenAI’s 4o-mini model. To complement the per-target latency evaluation, Table 7 summarizes the total time required to process 100 queries under identical experimental conditions. This analysis reflects expected throughput when scaling the LLM-based wellness prediction pipeline to larger user populations.

Among the three models, OpenAI 4o-mini provides the lowest latency (approximately 15 min per target) and the fastest throughput (102 s per 100 queries), making it well suited for near–real-time or interactive wellness applications. Gemini 2.0 Flash offers the most balanced trade-off between speed and cost, processing 100 queries in 197 s while reducing cost by roughly 40 percent relative to OpenAI. DeepSeek-Chat yields the lowest overall cost (approximately $0.07 per target) and moderate throughput (123 s per 100 queries), making it attractive for batch analytics and large-scale retrospective evaluations.

Despite the repeated bootstrap sampling, all models maintained per-target costs below $0.30, confirming the economic viability of LLM-based health inference pipelines. These results highlight a flexible deployment landscape: OpenAI excels in responsiveness, Gemini delivers strong cost–performance balance, and DeepSeek provides an efficient low-cost option for high-volume offline processing.

## 5. Ablation Study

To evaluate the robustness and generalizability of *HybridSense-LLM*, we conducted ablation analyses examining (A) feature-importance consistency across classical models, (B) sensitivity to temporal-window length, and (C) reproducibility across biomedical LLM architectures.

### 5.1. Feature-Importance Convergence

To assess the robustness of feature selection, we compared the top predictive features identified by Random Forest, XGBoost, and LASSO across all four wellness targets: fatigue, readiness, sleep quality, and stress. Despite their differing inductive biases, all three models consistently ranked mood-related variability descriptors, most notably Mood_skewness, Mood_std, Mood_cv, Mood_min, and Mood_rms, among the most influential predictors. This convergence indicates that short-term affective dynamics represent stable and model-independent signals for day-level wellness estimation.

Cardiovascular and sleep-related features, including Resting HR_cv, Resting HR_mean, Sleep Duration_max, and Sleep Duration_iqr, contributed more prominently to stress and sleep-quality prediction, whereas activity-driven metrics such as Calories Burn_p2p, Steps_p2p, and Steps_max were emphasized primarily by tree-based models, reflecting nonlinear behavioral effects. In contrast, LASSO favored sparse, linearly independent predictors such as Mood_iqr and Steps_cv, highlighting complementary linear relationships.

Overall, these results demonstrate strong cross-model agreement on key predictors while illustrating how relative feature salience is shaped by model-specific biases, with detailed importance scores reported in Tables S12–S15.

### 5.2. Temporal-Window Sensitivity

The main experiments in this study use 7-day rolling windows to ensure temporal stability and alignment with weekly behavioral patterns commonly observed in wearable data. To assess robustness to this design choice, we conducted a sensitivity analysis comparing 3-day and 5-day windows using GPT-4o-mini across seven prompting strategies. Shorter 3-day windows increased variability for readiness and fatigue, reflecting higher sensitivity to day-to-day fluctuations, whereas 5-day windows yielded more stable and accurate estimates for several targets. Across window lengths, zero-shot and zero-shot with self-consistency consistently achieved the lowest mean absolute errors, indicating strong numerical generalization without example-based supervision. Multi-step reasoning strategies improved interpretability but occasionally amplified variance for lower-signal targets such as sleep quality. Overall, these results indicate that while 7-day windows provide a principled and stable primary setting, moderate reductions in window length can further improve responsiveness for certain targets, highlighting a bias–variance tradeoff inherent in temporal aggregation (Tables S15 and S16).

### 5.3. Cross-Model Reproducibility

We further evaluated MedAlpaca-7B, an open-source biomedical LLM, under zero-shot prompting across five independent runs. The model exhibited highly consistent behavior, with standard errors below 0.01 for all targets. Mean MAE values were 2.57 for fatigue, 6.20 for readiness, 2.71 for sleep quality, and 2.87 for stress. These error levels are substantially higher than those achieved by HybridSense-LLM across all four wellness targets, indicating limited alignment between MedAlpaca’s biomedical pretraining and the structure of free-living wearable data. In contrast, HybridSense consistently yields lower error and tighter uncertainty bounds by leveraging structured statistical descriptors and hybrid signal representations tailored to wearable sensing. Together, these results demonstrate that performance differences are not driven by model instability or stochasticity, but by the suitability of the input representation and reasoning framework. This confirms that HybridSense-LLM maintains stable and robust inference behavior across diverse model families and operational conditions (Table 8).

## 6. Discussion

The findings of this study demonstrate that large language models can serve as effective reasoning engines for wellness prediction when supported by structured input representations. A direct comparison with prior work on PMData further highlights the strength of the proposed HybridSense framework. Health-LLM [12], the first LLM-based system evaluated on this dataset, reported higher error across stress, readiness, and sleep quality, even when using medically oriented models such as MedAlpaca, ClinicalCamel, BioMedGPT, and Asclepius. Relative to the strongest prompt-based Health-LLM configurations, HybridSense-LLM reduces error by approximately 39 percent for stress, 48 percent for readiness, and 32 percent for sleep quality, reflecting the value of combining engineered statistical descriptors with hybrid waveform context and structured prompting (Table 9). Fatigue is not directly comparable, as Health-LLM reports accuracy rather than MAE, but HybridSense produces stable and low error across all LLM families, contrasting with the high variability observed in the Health-LLM fatigue results. When compared with fine-tuned Health-LLM variants, the performance landscape becomes more nuanced: while fine-tuned models such as HealthAlpaca-13B achieve the lowest published MAEs for stress and sleep quality, HybridSense remains competitive despite requiring no task-specific training and clearly outperforms all fine-tuned baselines for readiness. Collectively, these results show that optimized input representation, statistical signal engineering, and hybrid waveform integration enable general-purpose LLMs to approach or exceed the performance of specialized, fine-tuned clinical models for wearable-based wellness prediction, while maintaining lower complexity, broader applicability, and zero training cost.

These results also align with developments in LLM-enabled clinical analytics. NYUTron demonstrated that system-scale language models trained on electronic health records can outperform traditional structured-data models for tasks such as readmission prediction, in-hospital mortality, and length of stay [11]. Other recent studies on personal health LLMs have explored integrating wearable data with conversational monitoring or behavioral coaching [22], reinforcing the growing role of generative AI in daily health decision support. Our framework contributes to this emerging ecosystem by showing that general-purpose LLMs can yield stable physiological inferences even without fine-tuning.

A key insight from our results is that the optimal prompting strategy depends strongly on the statistical properties of the target variable. For physiologically grounded targets such as fatigue and readiness, simple strategies like Zero-shot outperform multi-step reasoning methods. This is consistent with reprogramming-based findings from Time-LLM [13], which showed that LLMs generalize well from compact numerical summaries when tasks are formulated as direct input–output mappings. In contrast, subjective and noisy targets such as stress and sleep quality benefit from exemplar-based strategies (Few-shot, Few-shot+CoT) and ensembling approaches such as Self-Consistency. These patterns echo findings from multimodal reasoning studies, where providing statistical priors or example-driven scaffolds reduces the internal ambiguity of LLM predictions [16].

The HybridSense representation further strengthens model performance for systems capable of leveraging multimodal structure. DeepSeek-Chat, in particular, benefits from the inclusion of waveform segments, paralleling results from multimodal time-series forecasting models where hybrid numerical–textual inputs yield stronger representations than either modality alone [15]. Because LLMs do not inherently encode temporal dependencies, the combination of statistical descriptors with short contextual windows provides an efficient way to supply local temporal information without requiring sequence models.

The cost and latency analysis shows that LLM-driven physiological inference is feasible at scale. Even under repeated bootstrapping, per-target cost remained below $0.30 for all models. OpenAI 4o-mini offers the best responsiveness for near-real-time applications, while Gemini 2.0 Flash delivers strong economy, and DeepSeek-Chat provides the most cost-efficient pipeline for large offline batch analyses. As wearable sensor adoption continues to grow, with approximately 45% of U.S. adults using smartwatches or fitness trackers, LLM-based inference pipelines have the potential to support widespread, continuous wellness monitoring at very low operational cost [1].

### 6.1. Dataset Characteristics and Study Limitations

The PMData dataset used in this study is a publicly available multimodal wearable benchmark that combines continuous physiological and activity measurements with daily self-reported wellness labels. It includes data from 12 participants (9 male, 3 female), aged 25 to 60 years (mean age 34), collected over approximately three months. Available participant metadata are limited to basic demographic attributes such as age, gender, and height. Detailed clinical, behavioral, and lifestyle variables, including comorbidities, medication use, smoking status, menstrual phase, dietary intake, and supplement use, are not provided due to privacy and data-collection constraints. As a result, potential confounding factors known to influence physiological and emotional states cannot be explicitly modeled or controlled.

The wearable data consist of minute-level heart rate summaries rather than beat-to-beat recordings or standardized multi-minute segments. Accordingly, the proposed framework does not estimate clinical heart rate variability or autonomic nervous system function, which require specialized acquisition protocols and longer recording windows. All heart rate-derived features are therefore interpreted as coarse, day-level statistical descriptors that characterize overall cardiovascular activity patterns rather than physiological markers of autonomic regulation. The analyses are designed as within-subject, day-level evaluations of temporal associations between wearable-derived signals and subjective wellness states and are not intended for population-level, causal, or clinical inference.

The daily stress labels in PMData are collected using a simple 1 to 5 self-report scale designed for repeated, low-burden annotation in longitudinal wearable studies. This scale captures relative, day-level variations in perceived stress rather than clinically validated measures of stress or mental health. Accordingly, model outputs should be interpreted as within-subject estimates of perceived stress dynamics rather than diagnostic or clinical assessments. Evaluating the proposed framework using validated psychometric instruments remains an important direction for future work.

In addition, this study is based on a Fitbit-derived PMData dataset, and the results may not directly generalize to other wearable platforms with different sensing hardware, signal processing pipelines, or data availability. Extending the proposed framework to additional device ecosystems remains an important direction for future work.

The sample size is modest, and generalization to broader populations remains untested. Daily aggregation may mask intraday dynamics such as short-term stress spikes or sleep irregularities. In addition, models are evaluated independently; ensemble or fusion strategies may further improve performance. Future work will extend HybridSense to richer multimodal inputs and larger, more diverse cohorts.

Overall, these findings should be interpreted as exploratory evidence of how multimodal wearable data can be structured and reasoned over by large language models under realistic, unconstrained sensing conditions typical of consumer devices. Future work will require larger cohorts, richer demographic and clinical annotations, and higher-resolution physiological measurements to assess generalizability, disentangle confounding effects, and evaluate the clinical relevance of such frameworks.

### 6.2. Prompting Effects and Regression Behavior of Frontier LLMs

Our results indicate that the effectiveness of prompting strategies depends strongly on the statistical nature of the wellness target, reflecting intrinsic properties of frontier large language models rather than dataset-specific artifacts. Direct prompting (zero-shot) consistently achieves the lowest error for physiologically grounded targets such as fatigue and readiness across all evaluated model families. In contrast, multi-step reasoning strategies, including Chain-of-Thought, Tree-of-Thought, and Least-to-Most, frequently increase error for these targets, suggesting that intermediate textual reasoning can introduce additional variance when the objective is precise scalar prediction. This behavior is consistent with prior findings that frontier LLMs are trained via next-token prediction rather than explicit numerical loss minimization and therefore approximate regression through language generation rather than metric-optimized inference [13,42]. For more subjective and noisier outcomes, such as stress and sleep quality, exemplar-based and ensembling-style prompts (Few-shot, Self-Consistency) yield more stable performance by implicitly averaging over multiple generative trajectories [43]. These patterns persist across model families and temporal aggregation windows, indicating model-level inductive biases rather than preprocessing effects. A central aim of this study is to empirically examine how frontier LLMs handle classical regression-style tasks under different prompting regimes. A limitation is that we do not explore whether task-specific fine-tuning or hybrid numeric–symbolic training could further reduce variance in regression-oriented LLM inference.

Overall, this study positions large language models as a practical and extensible tool for wellness prediction from consumer wearables. By combining classical statistical signal processing with structured prompt design and hybrid input representations, HybridSense provides a scalable, interpretable, and computationally efficient framework for continuous wellness monitoring. Future work will extend this approach through larger cohorts, richer metadata, and higher-resolution physiological signals, enabling improved calibration and generalization. From a modeling perspective, this includes systematic prompt optimization, evaluation of parameter-efficient adaptation strategies, and integration of hybrid numeric–symbolic representations to better support regression-oriented reasoning in large language models. The modular architecture further facilitates integration with real-time wearable pipelines, supporting prospective evaluation of robustness, scalability, and deployment feasibility in real-world digital health settings.

## 7. Conclusions

This study presents HybridSense, a unified framework that combines engineered statistical descriptors with selective waveform context to enable large language models to perform accurate and interpretable wellness prediction from wearable-sensor data. Across multiple LLM families and prompting strategies, HybridSense delivers strong and reliable performance for readiness, stress, fatigue, and sleep quality, demonstrating that structured input representation is a critical driver of physiological inference. The results show that general-purpose LLMs, without any fine-tuning, can match or outperform prompt-based baselines from prior work and remain competitive with specialized fine-tuned clinical models, particularly for behaviorally coupled targets such as readiness. By integrating classical signal modeling with generative AI reasoning, HybridSense provides a scalable, training-free, and computationally efficient alternative to conventional time-series pipelines. This work positions LLM-enabled reasoning as a practical pathway for continuous, population-scale wellness monitoring and offers a blueprint for future multimodal and context-aware health analytics.

## Figures and Tables

**Table 1:** Summary of prompt engineering strategies evaluated in this study and their underlying reasoning mechanisms. Each method reflects a distinct way of guiding LLMs to interpret wearable-derived features, influencing both prediction stability and interpretability.

Method	Core Mechanism	Purpose
Zero-shot prompting	Direct prediction without examples	Uses pre-trained knowledge and generalization ability
Chain of Thought (CoT)	Produces reasoning steps	Improves interpretability and structured thinking
Tree of Thought (ToT)	Explores several reasoning paths	Finds the most plausible solution
Least to Most (L2M)	Solves smaller sub-problems in sequence	Encourages organized reasoning and reduces error
Self Consistency (SC)	Combines several independent outputs	Improves stability and reduces random variation
Few-shot prompting	Adds limited examples	Shows task format and mapping between input and output
Few-shot with CoT	Combines examples with reasoning	Supports both contextual learning and explanation

**Table 2:** Structured components of the prompt templates used in this study summarize the contextual, instructional, and formatting elements provided to the LLM.

Component	Description
Human persona	Defines the model’s role as a health-focused AI making the prediction.
Health Context	Explains how the input features relate to the wellness target.
Instruction	Provides required steps or remains empty if none are needed.
Example	A small example showing feature values and the correctly formatted output.
Output Format	Specifies the exact numeric response and required formatting.

**Table 3:** Top predictive features for fatigue, readiness, sleep quality, and stress. Mood-related features are included as contextual self-reported inputs rather than prediction targets.

Fatigue	Readiness	Sleep Quality	Stress
Mood_skewness	Mood_skewness	Mood_mean	Mood_cv
Mood_std	Mood_kurtosis	Mood_rms	Mood_min
Mood_cv	Calories Burn_p2p	Sleep Duration_max	Steps_skewness
Resting HR_skewness	Calories Burn_min	Resting HR_cv	Mood_std
Calories Burn_p2p	Mood_std	Sleep Duration_median	Mood_kurtosis
Calories Burn_kurtosis	Steps_mean	Resting HR_skewness	Mood_skewness
Mood_p2p	Steps_kurtosis	Steps_max	Sleep Duration_min
Sleep Duration_skewness	Mood_mean	Sleep Duration_iqr	Resting HR_min
Steps_kurtosis	Steps_p2p	Sleep Duration_skewness	Mood_rms
Sleep Duration_median	Sleep Duration_max	Resting HR_kurtosis	Calories Burn_cv

**Table 4:** Comparative Performance of Prompting Strategies Across Prediction Targets (MAE ± SE) Using OpenAI 4o-mini. StatFeature uses only preprocessed feature data derived from wearable sensors; HybridSense integrates both the engineered feature representations and the raw sensor waveforms.

Target	Method	StatFeature	HybridSense
Fatigue	Zero-shot	0.27 ± 0.00	0.20 ± 0.00
Fatigue	SC	0.45 ± 0.01	0.45 ± 0.01
Fatigue	CoT	1.31 ± 0.01	0.53 ± 0.01
Fatigue	ToT	0.63 ± 0.01	0.68 ± 0.01
Fatigue	L2M	1.56 ± 0.01	1.57 ± 0.01
Fatigue	Few-shot	0.64 ± 0.01	0.61 ± 0.02
Fatigue	Few-shot+CoT	0.60 ± 0.01	0.46 ± 0.00
Readiness	Zero-shot	0.55 ± 0.01	0.54 ± 0.01
Readiness	SC	0.73 ± 0.01	0.72 ± 0.01
Readiness	CoT	0.57 ± 0.01	1.22 ± 0.35
Readiness	ToT	0.57 ± 0.01	0.72 ± 0.01
Readiness	L2M	5.20 ± 0.02	1.03 ± 0.29
Readiness	Few-shot	2.92 ± 0.02	1.93 ± 0.28
Readiness	Few-shot+CoT	1.78 ± 0.01	2.89 ± 0.37
Sleep Quality	Zero-shot	1.38 ± 0.00	1.32 ± 0.06
Sleep Quality	SC	0.98 ± 0.01	0.92 ± 0.14
Sleep Quality	CoT	0.90 ± 0.01	0.58 ± 0.01
Sleep Quality	ToT	0.98 ± 0.01	0.53 ± 0.00
Sleep Quality	L2M	1.23 ± 0.01	1.55 ± 0.06
Sleep Quality	Few-shot	0.40 ± 0.01	0.25 ± 0.01
Sleep Quality	Few-shot+CoT	0.39 ± 0.01	0.58 ± 0.01
Stress	Zero-shot	0.39 ± 0.00	0.32 ± 0.00
Stress	SC	0.32 ± 0.01	0.24 ± 0.00
Stress	CoT	0.39 ± 0.01	0.67 ± 0.00
Stress	ToT	0.35 ± 0.00	0.33 ± 0.00
Stress	L2M	1.88 ± 0.00	1.88 ± 0.00
Stress	Few-shot	0.38 ± 0.00	0.27 ± 0.00
Stress	Few-shot+CoT	0.42 ± 0.01	0.71 ± 0.01

**Table 5:** Comparative Performance of Prompting Strategies Across Prediction Targets (MAE ± SE) Using Gemini 2.0 Flash. StatFeature uses only preprocessed feature data derived from wearable sensors; HybridSense integrates both the engineered feature representations and the raw sensor waveforms.

Target	Method	StatFeature	HybridSense
Fatigue	Zero-shot	0.22 ± 0.00	0.35 ± 0.01
Fatigue	SC	0.65 ± 0.01	0.39 ± 0.01
Fatigue	CoT	0.49 ± 0.01	1.45 ± 0.02
Fatigue	ToT	0.80 ± 0.01	0.51 ± 0.01
Fatigue	L2M	2.11 ± 0.01	1.21 ± 0.01
Fatigue	Few-shot	0.78 ± 0.00	0.31 ± 0.01
Fatigue	Few-shot+CoT	0.40 ± 0.01	0.76 ± 0.01
Readiness	Zero-shot	0.70 ± 0.01	0.46 ± 0.01
Readiness	SC	0.59 ± 0.01	0.85 ± 0.01
Readiness	CoT	0.88 ± 0.01	0.61 ± 0.01
Readiness	ToT	0.49 ± 0.01	1.05 ± 0.02
Readiness	L2M	4.51 ± 0.02	2.51 ± 0.30
Readiness	Few-shot	1.55 ± 0.01	3.50 ± 0.35
Readiness	Few-shot+CoT	3.21 ± 0.01	1.30 ± 0.28
Sleep Quality	Zero-shot	1.11 ± 0.00	0.88 ± 0.01
Sleep Quality	SC	0.61 ± 0.01	1.50 ± 0.12
Sleep Quality	CoT	1.26 ± 0.01	0.40 ± 0.01
Sleep Quality	ToT	0.70 ± 0.01	0.96 ± 0.01
Sleep Quality	L2M	1.51 ± 0.01	1.15 ± 0.01
Sleep Quality	Few-shot	0.59 ± 0.01	0.30 ± 0.00
Sleep Quality	Few-shot+CoT	0.30 ± 0.00	0.70 ± 0.01
Stress	Zero-shot	0.45 ± 0.01	0.25 ± 0.00
Stress	SC	0.29 ± 0.00	0.40 ± 0.01
Stress	CoT	0.50 ± 0.01	0.30 ± 0.00
Stress	ToT	0.30 ± 0.00	0.48 ± 0.01
Stress	L2M	1.50 ± 0.00	2.11 ± 0.01
Stress	Few-shot	0.25 ± 0.00	0.51 ± 0.01
Stress	Few-shot+CoT	0.55 ± 0.01	0.35 ± 0.01

**Table 6:** Comparative Performance of Prompting Strategies Across Prediction Targets (MAE ± SE) Using DeepSeek-Chat. StatFeature uses only preprocessed feature data derived from wearable sensors; HybridSense integrates both the engineered feature representations and the raw sensor waveforms.

Target	Method	StatFeature	HybridSense
Fatigue	Zero-shot	0.45 ± 0.01	0.34 ± 0.00
Fatigue	SC	0.87 ± 0.01	0.67 ± 0.01
Fatigue	CoT	0.76 ± 0.02	0.59 ± 0.02
Fatigue	ToT	0.86 ± 0.01	0.66 ± 0.01
Fatigue	L2M	1.43 ± 0.01	1.10 ± 0.01
Fatigue	Few-shot	0.49 ± 0.01	0.38 ± 0.01
Fatigue	Few-shot+CoT	0.83 ± 0.01	0.64 ± 0.01
Readiness	Zero-shot	1.02 ± 0.03	0.73 ± 0.02
Readiness	SC	1.27 ± 0.04	0.91 ± 0.03
Readiness	CoT	1.32 ± 0.03	0.94 ± 0.02
Readiness	ToT	1.10 ± 0.03	0.78 ± 0.02
Readiness	L2M	1.11 ± 0.03	0.79 ± 0.02
Readiness	Few-shot	2.03 ± 0.04	1.45 ± 0.03
Readiness	Few-shot+CoT	2.48 ± 0.04	1.77 ± 0.03
Sleep Quality	Zero-shot	0.58 ± 0.01	0.49 ± 0.01
Sleep Quality	SC	0.84 ± 0.02	0.70 ± 0.02
Sleep Quality	CoT	0.63 ± 0.00	0.53 ± 0.00
Sleep Quality	ToT	0.79 ± 0.03	0.66 ± 0.03
Sleep Quality	L2M	0.86 ± 0.01	0.72 ± 0.01
Sleep Quality	Few-shot	0.23 ± 0.00	0.19 ± 0.00
Sleep Quality	Few-shot+CoT	0.47 ± 0.01	0.39 ± 0.01
Stress	Zero-shot	0.40 ± 0.01	0.32 ± 0.01
Stress	SC	0.40 ± 0.01	0.32 ± 0.00
Stress	CoT	1.01 ± 0.02	0.81 ± 0.02
Stress	ToT	0.26 ± 0.00	0.21 ± 0.00
Stress	L2M	2.34 ± 0.01	1.87 ± 0.00
Stress	Few-shot	0.37 ± 0.01	0.30 ± 0.01
Stress	Few-shot+CoT	0.90 ± 0.01	0.72 ± 0.01

**Table 7:** Per-Target Computational Cost and Latency for LLM Inference (30 Bootstrap Iterations per Target).

Model	Average Cost (USD)	Average Latency (Minutes)	Total Inference Time (Seconds per 100 Queries)
ChatGPT (OpenAI 4o-mini)	$0.26	15	102
Gemini 2.0 Flash	$0.15	20	197
DeepSeek-Chat	$0.07	30	123

**Table 8:** Zero-shot performance of fine-tuned biomedical large language models on PMData. The table reports mean MAE ± standard error for MedAlpaca-7B and BioMistral-7B across four wellness targets.

Model	Target	MAE (Mean ± SE)
MedAlpaca-7B	Stress	2.87 ± 0.01
MedAlpaca-7B	Sleep Quality	2.71 ± 0.01
MedAlpaca-7B	Readiness	6.20 ± 0.01
MedAlpaca-7B	Fatigue	2.57 ± 0.01
BioMistral-7B	Fatigue	0.21 ± 0.00
BioMistral-7B	Readiness	0.52 ± 0.01
BioMistral-7B	Sleep Quality	1.36 ± 0.02
BioMistral-7B	Stress	0.34 ± 0.01

**Table 9:** Comparative prompt-based performance of our proposed HybridSense-LLM and Health-LLM on the PMData dataset.

Target	Health-LLM Best Performance (MAE)	HybridSense-LLM Best Performance (MAE)	Relative Improvement (%)
Stress	0.33 (CoT-SC, GPT-4)	0.20 (ZS with OpenAI 4o-mini)	39.4% ↓
Readiness	0.86 (CoT-SC, GPT-4)	0.45 (ZS, Gemini 2.0 Flash)	47.7% ↓
Sleep Quality	0.37 (ZS, ClinicalCamel)	0.250 (Few-shot, OpenAI 4o-mini)	32.4% ↓
Fatigue	77.0% (CoT, GPT-3.5)	0.20 (ZS, OpenAI 4o-mini)	Not comparable ^1^

^1^ Health-LLM reports fatigue as an accuracy-like metric , while HybridSense uses MAE (↓ = better). Direct percentage comparison is not meaningful.

## Data Availability

Data is contained within the article or Supplementary Materials.

## Supplementary Materials

The following supporting information can be downloaded at: https://www.mdpi.com/article/10.3390/bioengineering13010120/s1. Tables S1–S4 include additional dataset descriptions, preprocessing details, and feature definitions. Tables S5–S12 provide the full prompt templates and instruction formats. Tables S13–S14 report feature importance analyses across wellness targets using XGBoost and LASSO models, respectively. Tables S15–S16 present temporal window sensitivity analyses comparing 5-day and 3-day sliding windows for wellness prediction performance across multiple prompting strategies.
