# Supplementary material for: HybridSense-LLM: A Structured Multimodal Framework for Large-Language-Model–Based Wellness Prediction from Wearable Sensors with Contextual Self-Reports
Source: Bioengineering (Basel). 2026 Jan 20;13(1):120. doi: 10.3390/bioengineering13010120 (PMC12837951; doi:10.3390/bioengineering13010120)
Supplement: Supplementary file 1 [file bioengineering-13-00120-s001.zip › bioengineering-4043768-supplementary.pdf]

HybridSense-LLM: A Structured Multimodal Framework for Large Language Model–Based Wellness Prediction from Wearable Sensors with Contextual Self-Reports

Cheng-Huan Yu[1], and Mohammad Masum[2, *]

This document provides supplementary tables supporting the main manuscript, including dataset characteristics, preprocessing details, statistical feature definitions, and full prompt templates for all prompting strategies evaluated.

Table S1: The table summarizes five key physiological and behavioral signals collected from wearable devices and self-reports, detailing how each is transformed into daily predictors for wellness estimation.

Signal	Source	Native cadence	Analysis cadence	Unit
Steps	Fitbit activity log	5 min (event log)	Daily (sum)	steps/day
Calories burned	Fitbit activity log	5 min (event log)	Daily (sum)	kcal/day
Resting heart rate	Fitbit summary	Daily	Daily (mean)	bpm
Sleep duration	Fitbit sleep summary	Nightly	Daily (hours)	hours/day
Mood	Self-report (Likert)	Daily	Daily (score)	1–5

Table S2: This table provides an overview of participant characteristics, data volume, feature composition, and preprocessing steps, highlighting how heterogeneous raw logs were standardized into a consistent analytical dataset for wellness prediction.

Item	Value
Participants	12
Total daily records	1836
Aggregation frequency	Daily (uniform)
Sensors and logs	Heart rate, step count, activity, sleep
Targets (labels)	Readiness (0–10), Stress (0–5), Fatigue (0–5), Sleep quality (0–5)
Features per record	About 25–30 numerical descriptors (mean, std, skewness, kurtosis, RMS, IQR…)

Table S3: Illustrative harmonized daily records after aligning wearable-derived features with corresponding self-reported wellness labels. Sliding-window exclusion; any 7-day window with missing daily records is discarded. No statistical imputation is applied.

PID	Date	HR	Step	…	Readiness	Stress	Fatigue	Sleep Quality
P01	2024-05-12	72.4	9.8	…	2	1	4	2
P01	2024-05-13	62.4	7.2	…	1	3	4	3
…	…	…	…	…	…	…	…	…
P07	2024-06-03	65.1	7.2	…	3	2	3	4
P07	2024-06-19	78.6	11.3	…	1	1	5	4

Table S4: Statistical descriptors used to summarize wearable-derived signals at the day and window level.

Feature	Formula	Description
Mean	$\mu = \frac{1}{N}\sum_{i=1}^{N} x_i$	Average value
STD	$\sigma = \sqrt{\frac{1}{N}\sum_{i=1}^{N}(x_i - \mu)^2}$	Standard deviation
Max	$\max(x_i)$	Maximum value
Min	$\min(x_i)$	Minimum value
P2P	$\max(x_i) - \min(x_i)$	Peak-to-peak range
RMS	$\sqrt{\frac{1}{N}\sum_{i=1}^{N} x_i^2}$	Root mean square
Skewness	$\frac{1}{N}\sum_{i=1}^{N}\frac{(x_i - \mu)^3}{\sigma^3}$	Asymmetry of the distribution
Kurtosis	$\frac{1}{N}\sum_{i=1}^{N}\frac{(x_i - \mu)^4}{\sigma^4}$	Tail heaviness
CV	$\frac{\sigma}{\mu}$	Coefficient of variation
IQR	$Q_{75} - Q_{25}$	Interquartile range
Median	Q_{50}	50th percentile

Table S5: Zero-shot prompt structure for estimating daily stress levels.

**Component**	**Prompt Content**
Human persona	You are a health-focused AI model trained to estimate daily stress levels using physiological and behavioral indicators.<br>Your goal is to predict a stress score (a real number between 0 and 5) using the features below.
Health Context	Input features: Mood_skewness: -1.78, Mood_std: 0.37, Mood_cv: 0.13,
Instruction	Empty
Output Format	Output format:<br>- Output a single real number between 0 and 5.<br>- Do not provide any explanation or reasoning. Output format: Stress level is x.x Only this format.

Table S6: CoT prompt structure for estimating daily stress levels.

Component	Prompt Content
Human persona	You are a health-focused AI model trained to estimate daily stress levels using physiological and behavioral indicators.<br>Your goal is to predict a stress score (a real number between 0 and 5) using the features below.
Health Context	Input features: Mood_skewness: -1.78, Mood_std: 0.37, Mood_cv: 0.13,
Instruction	1) Think step by step internally (do not reveal your reasoning).<br>2) First, analyze the statistics of the key features provided.<br>3) Reason internally how each may reflect low, moderate, or high stress.<br>4) Integrate your reasoning to estimate the overall stress level.
Output Format	Output format:<br>- Output a single real number between 0 and 5.<br>- Do not provide any explanation or reasoning. Output format: Stress level is x.x Only this format.

Table S7: Self-Consistency prompt structure for estimating daily stress levels.

Component	Prompt Content
Human persona	You are a health-focused AI model trained to estimate daily stress levels using physiological and behavioral indicators.<br>Your goal is to predict a stress score (a real number between 0 and 5) using the features below.
Health Context	Input features: Mood_skewness: -1.78, Mood_std: 0.37, Mood_cv: 0.13,
Instruction	1. Analyze the statistical patterns of each feature carefully.<br>2. Internally reason step by step about how these patterns may reflect low, moderate, or high stress.<br>3. Use self-consistency:<br>- Internally generate 11 diverse reasoning paths (with different initial assumptions or sampling).<br>- For each path, produce an internal provisional score si in [0, 5].<br>- Aggregate by taking the median of si, then clip to [0,5] and round to one decimal place.
Output Format	Output format:<br>- Output a single real number between 0 and 5.<br>- Do not provide any explanation or reasoning. Output format: Stress level is x.x. Only this format.

Table S8: ToT prompt structure for estimating daily stress levels.

Component	Prompt Content
Human persona	You are a health-focused AI model trained to estimate daily stress levels using physiological and behavioral indicators.<br>Your goal is to predict a stress score (a real number between 0 and 5) using the features below.
Health Context	Input features: Mood_skewness: -1.78, Mood_std: 0.37, Mood_cv: 0.13,
Instruction	Instructions (Tree-of-Thought reasoning):<br>- Step 1: Generate at least three independent reasoning paths based on different combinations or interpretations of the features above.<br>- Step 2: Evaluate the stress level under each reasoning path.<br>- Step 3: Compare the outputs from the three branches.<br>- Step 4: Choose the most consistent or averaged outcome as the final decision
Output Format	Output format:<br>- Output a single real number between 0 and 5.<br>- Do not provide any explanation or reasoning. Output format: Stress level is x.x. Only this format.

Table S9: L2M prompt structure for estimating daily stress levels.

Component	Prompt Content
Human persona	You are a health-focused AI model trained to estimate daily stress levels using physiological and behavioral indicators.<br>Your goal is to predict a stress score (a real number between 0 and 5) using the features below.
Health Context	Input features: Mood_skewness: -1.78, Mood_std: 0.37, Mood_cv: 0.13,
Instruction	Instructions:<br>1. Based on the mood-related variability features, what is the stress indication from the mood?<br>2. Based on the sleep pattern descriptors, what is the stress indication from sleep?<br>3. Based on the activity metrics, what is the stress indication from physical activity?<br>4. Based on your answers above, estimate the final stress score.
Output Format	Output format:<br>- Output a single real number between 0 and 5.<br>- Do not provide any explanation or reasoning. Output format: Stress level is x.x. Only this format.

Table S10: Few-Shot prompt structure for estimating daily stress levels.

Component	Prompt Content
Human persona	You are a health-focused AI model trained to estimate daily stress levels using physiological and behavioral indicators.<br>Your goal is to predict a stress score (a real number between 0 and 5) using the features below.
Health Context	Input features: Mood_skewness: -1.78, Mood_std: 0.37, Mood_cv: 0.13,
Instruction	Empty
Example	Mood_skewness: -1.08, Mood_std: 1.37, Mood_cv:0.80; Stress level is 3.72".<br>Mood_skewness: 1.38, Mood_std: 0.73, Mood_cv:0.57; Stress level is 2.27".<br>Mood_skewness: 0.75, Mood_std: 1.13, Mood_cv:0.32; Stress level is 3.12".
Output Format	Output format:<br>- Output a single real number between 0 and 5.<br>- Do not provide any explanation or reasoning. Output format: Stress level is x.x. Only this format.

Table S11: Few-Shot+Cot prompt structure for estimating daily stress levels.

Component	Prompt Content
Human persona	You are a health-focused AI model trained to estimate daily stress levels using physiological and behavioral indicators.<br>Your goal is to predict a stress score (a real number between 0 and 5) using the features below.
Health Context	Input features: Mood_skewness: -1.78, Mood_std: 0.37, Mood_cv: 0.13,
Instruction	Instructions:<br>1) Think step by step internally (do not reveal your reasoning).<br>2) First, analyze the statistics of the key features provided.<br>3) Reason internally how each may reflect low, moderate, or high stress.<br>4) Integrate your reasoning to estimate the overall stress level.
Example	Mood_skewness: -1.08, Mood_std: 1.37, Mood_cv:0.80; Stress level is 3.72".<br>Mood_skewness: 1.38, Mood_std: 0.73, Mood_cv:0.57; Stress level is 2.27".<br>Mood_skewness: 0.75, Mood_std: 1.13, Mood_cv:0.32; Stress level is 3.12".
Output Format	Output format:<br>- Output a single real number between 0 and 5.<br>- Do not provide any explanation or reasoning. Output format: Stress level is x.x. Only this format.

Table S12: **Random Forest feature importance across wellness targets.** Impurity-based importance scores (mean decrease in variance) are reported for the top ten predictive features for each wellness target. Values in parentheses indicate normalized importance scores, reflecting the relative contribution of each feature to prediction accuracy.

**Target**	**Top-10 Predictive Features** by RF
Fatigue	Mood_skewness (0.14), Mood_std (0.13), Mood_cv (0.12), Calories Burn_p2p (0.09), Resting HR_skewness (0.08), Calories Burn_kurtosis (0.07), Mood_p2p (0.07), Sleep Duration_skewness (0.06), Steps_kurtosis (0.06), Sleep Duration_median (0.05)
Readiness	Mood_skewness (0.15), Mood_kurtosis (0.12), Calories Burn_p2p (0.11), Calories Burn_min (0.10), Mood_std (0.10), Steps_mean (0.09), Steps_kurtosis (0.08), Mood_mean (0.07), Steps_p2p (0.07), Sleep Duration_max (0.06)
Sleep Quality	Mood_mean (0.14), Mood_rms (0.13), Sleep Duration_max (0.16), Resting HR_cv (0.11), Sleep Duration_median (0.10), Steps_max (0.09), Sleep Duration_iqr (0.09), Sleep Duration_skewness (0.08), Resting HR_skewness (0.07), Resting HR_kurtosis (0.06)
Stress	Mood_cv (0.17), Mood_min (0.12), Steps_skewness (0.11), Mood_std (0.10), Mood_kurtosis (0.09), Mood_skewness (0.09), Sleep Duration_min (0.08), Resting HR_min (0.07), Mood_rms (0.07), Calories Burn_cv (0.06)

Table S13. **XGBoost feature importance across wellness targets.**Feature importance scores based on gain are shown for the top ten predictors for each wellness target. Values in parentheses denote gain-based importance scores, representing the average reduction in loss attributable to splits using each feature.

**Target**	**Top-10 Predictive Features** by XGBoost
Fatigue	Mood_skewness (0.19), Mood_std (0.16), Mood_cv (0.14), Calories Burn_p2p (0.10), Calories Burn_kurtosis (0.09), Resting HR_skewness (0.08), Mood_p2p (0.08), Sleep Duration_skewness (0.07), Steps_kurtosis (0.06), Sleep Duration_median (0.06)
Readiness	Mood_min (0.17), Mood_kurtosis (0.14), Steps_p2p (0.12), Calories Burn_min (0.11), Mood_std (0.10), Steps_mean (0.09), Mood_mean (0.08), Steps_kurtosis (0.08), Sleep Duration_max (0.07), Calories Burn_p2p (0.06)
Sleep Quality	Sleep Duration_max (0.19), Mood_mean (0.14), Resting HR_cv (0.11), Sleep Duration_iqr (0.10), Steps_max (0.09), Sleep Duration_median (0.09), Sleep Duration_skewness (0.08), Resting HR_skewness (0.07), Resting HR_kurtosis (0.06), Mood_rms (0.06)
Stress	Mood_cv (0.20), Mood_std (0.13), Steps_skewness (0.11), Mood_min (0.10), Mood_kurtosis (0.09), Mood_skewness (0.09), Sleep Duration_min (0.08), Resting HR_min (0.07), Mood_rms (0.07), Calories Burn_cv (0.06)

Table S14. LASSO feature importance across wellness targets. Feature importance is quantified using the absolute values of standardized regression coefficients. The top ten predictors for each wellness target are listed, with values in parentheses rounded to two decimal places, highlighting sparse and linearly independent contributions.

**Target**	**Top-10 Predictive Features** by LASSO
Fatigue	Mood_iqr (0.18), Steps_cv (0.12), Mood_std (0.11), Calories Burn_cv (0.10), Steps_p2p (0.09), Sleep Duration_iqr (0.08), Resting HR_cv (0.07), Mood_mean (0.07), Steps_mean (0.06), Calories Burn_mean (0.06)
Readiness	Mood_iqr (0.20), Calories Burn_min (0.11), Steps_cv (0.10), Mood_std (0.09), Sleep Duration_max (0.08), Resting HR_cv (0.08), Steps_mean (0.07), Calories Burn_mean (0.07), Mood_mean (0.06), Sleep Duration_iqr (0.06)
Sleep Quality	Sleep Duration_iqr (0.21), Resting HR_cv (0.10), Mood_iqr (0.09), Steps_cv (0.08), Mood_std (0.08), Calories Burn_cv (0.07), Steps_mean (0.07), Mood_mean (0.06), Sleep Duration_mean (0.06), Resting HR_mean (0.05)
Stress	Mood_min (0.23), Mood_cv (0.14), Steps_cv (0.10), Mood_std (0.09), Calories Burn_cv (0.08), Resting HR_cv (0.08), Mood_mean (0.07), Steps_mean (0.07), Sleep Duration_iqr (0.06), Calories Burn_mean (0.06)

Table S15. Temporal window sensitivity analysis using a 5-day sliding window. Mean absolute error (MAE) values are reported for fatigue, readiness, sleep quality, and stress prediction using GPT-4o-mini across seven prompting strategies. Results quantify prediction accuracy when daily wellness scores are aggregated over 5-day windows.

Method	Fatigue	Readiness	Sleep Quality	Stress
Zero-Shot (ZS)	0.21	0.54	1.42	0.32
ZS-SC	0.42	0.76	1.39	0.26
FS	0.62	1.14	0.35	0.27
FS-CoT	0.48	1.19	0.28	0.70
CoT	0.57	0.56	0.94	0.71
TOT	0.64	0.69	1.02	0.33
L2M	1.56	0.81	1.30	1.86

Table S16. Temporal window sensitivity analysis using a 3-day sliding window. Mean absolute error (MAE) values are reported for fatigue, readiness, sleep quality, and stress prediction using GPT-4o-mini across seven prompting strategies. Results quantify prediction accuracy under shorter temporal aggregation and enable direct comparison with the 5-day window analysis.

Method	Fatigue	Readiness	Sleep Quality	Stress
ZS	0.32	0.77	1.37	0.29
ZS-SC	0.42	0.77	1.39	0.26
COT	0.67	0.79	1.13	0.69
TOT	0.38	0.69	0.78	0.31
FS	0.66	1.65	0.39	0.36
FS-COT	0.48	1.19	0.28	0.71
L2M	1.56	0.81	1.30	1.06
